# Drying Technique Providing Maximum Benefits on Hydrogelling Ability of Avocado Seed Protein: Spray Drying

**DOI:** 10.3390/foods12234219

**Published:** 2023-11-22

**Authors:** Bakhtiyar Azad Abdullah, Bulent Basyigit, Mehmet Karaaslan

**Affiliations:** 1 Department of Biology, Faculty of Science and Health, Koya University, Danielle Mitterrand Boulevard, Koya KOY45, Kurdistan Region—F.R., Iraq; 2 Food Engineering Department, Engineering Faculty, Harran University, Sanliurfa 63000, Turkey; bulentbasyigit@harran.edu.tr (B.B.); mk385@cornell.edu (M.K.)

**Keywords:** avocado seed protein, drying techniques, natural hydrogel, release behavior

## Abstract

The current study focused on creating natural hydrogels consisting of mixtures of avocado seed proteins dried with different techniques and locust bean gum. Proteins were extracted from avocado seed by alkali and isoelectric precipitation methods. Avocado seed proteins were dried by five different drying methods, namely ambient drying, oven drying, vacuum drying, freeze drying, and spray drying. FT-IR spectra were used to analyze the chemical structure of proteins dried using various techniques. Additionally, hydrogel models were constructed in the presence of avocado seed proteins and locust bean gum to clarify the effect of drying techniques on their hydrogelling ability. The impact of drying techniques on the functional behavior of hydrogels was notable. The maximum water holding capacity values were detected in the hydrogel system containing spray-dried proteins (93.79%), followed by freeze-dried (86.83%), vacuum-dried (76.17%), oven-dried (72.29%), and ambient-dried (64.8%) counterparts. The swelling ratio was 34.10, 33.51, 23.05, 18.93, and 14.39% for gels in the presence of freeze-dried, spray-dried, vacuum-dried, oven-dried, and ambient-dried proteins, respectively. Additionally, the desirable values for the amount of protein leaking from the systems prepared using spray-dried (7.99%) and freeze-dried (12.14%) proteins were obtained compared to others (ambient-dried: 24.03%; oven-dried: 17.69%; vacuum-dried: 19.10%). Superior results in terms of textural properties were achieved in hydrogel models containing spray-dried and freeze-dried proteins. In general, hydrogel models exhibited elastic behavior rather than viscous properties; however, the magnitudes of elasticity varied. Furthermore, the success of gels containing hydrogel models containing spray-dried protein and locust bean gum in the bioactive compound delivery system was obvious compared with protein ones alone.

## 1. Introduction

Gels possess a unique physical state that exhibits characteristics of both solids and liquids, rendering them neither fully solid nor fully liquid. These entities serve as an intermediary state between these two forms and demonstrate characteristics of both elasticity and fluidity. The predominant composition of food materials in the realm of design consists of gels. These gels are typically constructed using hydrocolloids, specifically proteins and polysaccharides, which serve as the fundamental building blocks in their manufacturing process [1,2]. Food gels could be categorized as soft solid materials, with the aqueous phase constituting over 80% of their overall structure [3]. Gels such as jam, jelly, confectionery items, desserts, and quick-set gels exemplify this category. In recent years, there has been an observed inclination towards the utilization of these unique structures. This may be attributed to their notable attributes such as high water content, biodegradability, appealing taste, affordability, low-calorie content, biocompatibility, and satiety-enhancing properties [1,4,5]. Hydrogels are polymeric networks that have a three-dimensional structure with interconnected chains that possess hydrophilic properties. They could be fabricated using synthetic (acrylic acid, poly (vinyl alcohol), and so on) and natural (protein and polysaccharides) materials [6]. The distinctive attributes of both systems are associated with their exceptional water-absorption qualities (non-dissolution in aqueous environments) and their capacity to undergo significant swelling without compromising their structural integrity [7]. Recently, hydrogels have been used in various systems, namely, food applications, encapsulation, delivery systems, calorie management, and food packaging are a few examples [8]. A prior work documented the application of hydrogel systems for the encapsulation of phenolic chemicals [9]. Likewise, the efficacy of these structures has been proven in the transportation mechanisms of phenolic and aromatic chemicals [10]. Synthetic hydrogel systems are ahead in terms of the mentioned characteristic properties compared with natural counterparts, indicating that they possess more widespread usage in applications. However, various disadvantages (toxicity and non-environmentally friendly) associated with these systems were reported by previous studies [11]. Therefore, in recent years, there has been an increase in the number of studies involving the development of natural hydrogels (especially protein-based ones) and their incorporation into different systems.

The global issue of protein deficiency is significantly increasing and affecting both developing and developed countries. This problem is mostly attributed to population growth, as well as the rising demand for protein in aquaculture, feed production, and industrial applications [12]. Even though plants, animals and microorganisms are available protein sources, their capabilities cannot meet total protein demand. Therefore, the utilization of by-products or waste materials for protein extraction is regarded as a viable, valuable, economical and replicable approach to augmenting protein availability. One of the primary obstacles encountered in the process of protein extraction from waste resources is efficiently eliminating the contaminants while simultaneously enhancing protein production, reducing extraction duration, and preserving the functional attributes of the proteins [13].

The avocado (*Persea Americana Mill*) plant is a dicotyledonous plant from the family Lauraceae, belonging to the plant-flowering family and originating from Mexico and Central America. Avocado fruits are among the most widely consumed fruits on a global scale [14]. During the processing industry, avocado seeds are considered a waste by-product and discarded. This by-product is causing environmental pollution because it has not been utilized [15]. Waste by-products could be beneficial in terms of the economy and environment by good management. This is because avocado seeds constitute a significant fraction (13–17%) and it is rich in a variety of functional and bioactive compounds, including protein, crude fiber, polysaccharides, vitamins, lipids, and minerals abundant in phytochemicals [16,17]. Protein is a significant constituent among the assortment of macromolecules present in avocado seeds. Proteins are macromolecules of significant complexity, composed of amino acids, which fulfill crucial functions in various biological processes such as growth, cellular communication, enzymatic control, and bio-catalysis [18]. The growing demand for nutritionally better food has led to greater interest in plant-based nutrients, particularly protein. Consequently, significant attention has been devoted to the exploration of sustainable alternatives for nutritionally dense food sources.

Nowadays, protein is extracted from different fruit seeds by using different extraction methods and drying by different drying techniques. The functional properties and qualities of proteins derived from various plant sources exhibit differences in terms of solubility, gelation, water/oil absorption capacity, emulsion stability, and foam stability. These variations could be attributed to the diverse structure and composition of proteins. The functional properties of proteins are major factors in evaluating the quality of protein, which depends on the extraction methods and drying techniques. According to previous studies, spray drying, freeze drying, vacuum drying, and oven drying are the most widely used drying techniques in food protein processing [19]. The variations in temperature and retention time throughout the drying process might lead to alterations in the physicochemical characteristics and functional properties of proteins [20]. Consequently, these changes could give rise to diverse functional qualities in the resultant dried products. Previously, some studies have reported that drying techniques have a remarkable effect on the physiochemical characteristic and functional properties of mung bean, quinoa, hempseed, chia, and fenugreek isolation protein powders, and the results illustrated the differences in physicochemical and functional properties of different drying methods [19,20,21,22,23]. Additionally, the drying technique had a considerable impact on the characteristic properties (morphological structure and lipophilic components) of mushroom (*Pleurotus ostreatus*) powder, which was highlighted elsewhere [24].

The objective of this study was to investigate the extraction of proteins from avocado seed and drying protein with five different drying methods, namely ambient drying (AD), oven drying (OD), vacuum drying (VD), freeze-drying (FD), and spray drying (SD). Following that, the effect of drying techniques on the chemical composition and FT-IR spectra of avocado seed protein (ASP) were evaluated. Additionally, the effect of drying techniques on the hydrogelling ability of ASP was investigated. For these, hydrogel systems of five different natures were produced. These were systems prepared in the presence of ambient-dried avocado seed protein with locust bean gum (LB) (AD-ASP + LB), oven-dried avocado seed protein with LB (OD-ASP + LB), vacuum-dried avocado seed protein with LB (VD-ASP + LB), freeze-dried avocado seed protein with LB (FD-ASP + LB), and spray-dried avocado seed protein with LB (SD-ASP + LB). Functional properties (water holding capacity (WHC), swelling ratio, and protein leachability) and mechanical behaviors (textural and rheological) of hydrogels were analyzed. Furthermore, one of the aims was to prove how effective hydrogel systems were at delivering bioactive compounds.

## 2. Materials and Methods

### 2.1. Materials

Avocado seeds were provided from the main production area in Mersin province of Turkey. LB was purchased from Öz Yildiz Chemical Industry Trade Ltd., Co. (Istanbul, Turkey). NaOH, HCl, NaCl, and hexane were supplied from Merck (Darmstadt, Germany). NaH_2_PO_4_ and Na_2_HPO_4_ were provided by Sigma-Aldrich (St. Louis, MO, USA). All chemicals and solvents were standard analytical grade.

### 2.2. Production

#### 2.2.1. Defatting Avocado Seed

The production of oil-free avocado seeds was conducted according to previous studies with some modifications [25,26]. Avocado seeds were ground by a laboratory grinder (DMS 253, Demsan, Turkey) and then dried using a cabinet-type dryer (Nüve EN120, Nüve, Ankara, Turkey) at 45 °C for 24 h. For the defatting process, the Soxhlet extraction procedure was applied. For this, 20 g avocado seed was placed in a 30 mm × 200 mm cellulose thimble and 200 mL hexane was added into the main chamber of a 250 mL Soxhlet extractor equipped with a condenser. The temperature was adjusted to the boiling point of the solvent (65 °C), and the extraction process was conducted for 120 min under reflux conditions. At the end of the process, after hexane was evaporated at 110 °C, the defatting seeds were collected into plastic bags (20 cm × 30 cm (W × H) and 0.05 mm thickness) and stored at 4 °C for future analyses.

#### 2.2.2. Protein Extraction from Avocado Seed

Protein was extracted from defatted avocado seeds by using the alkali extraction-isoelectric precipitation method as described in a previous study [27] with slight modifications. Briefly, 10 g of defatted avocado seed was mixed with 100 mL of 0.12 mol/L NaOH. The extraction process was maintained in a Lab-Line incubator-shaker (Lab-Line Instruments, Inc., Melrose Park, IL, USA) at 250 rpm for 1 h at 37 °C. Following that, the protein solution was centrifuged (Nüve NF615, Nüve, Ankara, Turkey) at 4000 rpm for 15 min at 4 °C. Next, the supernatant was collected and adjusted pH to 4.5 with 1 mol/L HCl by a Hanna Instruments Edge^®®^ Blue HI 2202–02 pH meter (Woonsocket, RI, USA) before being kept in the refrigerator at 4 °C overnight. Finally, the extracted proteins were dried using five different drying methods to obtain powder, as follows.

#### 2.2.3. Drying methods

Ambient drying (AD): protein solutions were transferred to plastic plates with 5 mm depth, and the drying process was performed for 96 h at room temperature (~25 °C).

Oven drying (OD): protein solutions were dried by a cabinet-type dryer (Nüve EN120, Nüve, Ankara, Turkey) at 50 °C for 24 h.

Vacuum drying (VD): extracted proteins were dried by a BINDER VD 23 vacuum drying oven (BINDER, New York, NY, USA) at 50 °C for 24 h under pressure with a value of 85 Kpa.

Freeze drying (FD): Protein solutions were frozen at −18 °C for 18 h. Next, samples were placed into a freeze dryer (Armfield SB4, Ringwood, UK) and dried at −55 °C compressor temperature for 24 h under vacuum pressure (20 Pa). 

Spray drying (SD): Protein solutions were diluted with deionized water at (1:2 *v*/*v*) and fed to a spray dryer (Unopex B15, Bornova İzmir, Turkey). The inlet temperature and feeding rate were adjusted to 140 °C and 6 mL/min, respectively.

Powdered proteins were transferred to plastic bags (20 cm × 30 cm (W × H) and 0.05 mm thickness) and stored in a refrigerator at 4 °C for future analyses.

#### 2.2.4. Hydrogel Preparation

The solutions of ASP (24%, *w*/*v*) and LB (2%, *w*/*v*) were prepared separately under continuous shaking at 150 rpm for 5 h at room temperature (~25 °C). Solutions were kept at 4 °C to provide complete hydration overnight. ASP and LB solutions were mixed at a ratio of 1:1 (*v*/*v*), and the mixture process was performed for 2 h at 150 rpm in a Lab-Line incubator-shaker. The pH of the final solutions was adjusted to 7.0. Solutions were transferred to a water bath (Nüve St 402, Nüve, Ankara, Turkey) and kept at 90 °C for 50 min. Samples cooled to room temperature were held at 4 °C for future analysis [28].

### 2.3. Characterization and Technofunctional Analyses

#### 2.3.1. Avocado Seed Protein Analyses

##### Chemical Composition

Protein content was determined by using combustion nitrogen analysis [29]. Moisture content, lipid content, ash content, and water activity analyses of ASPs were performed according to a previous method [27]. 

##### Fourier Transform Infrared Spectroscopy

The chemical structure of ASP was determined by using FT-IR spectroscopy (FT-IR IRTracer-100, Shimadzu Co., Kyoto, Japan). Spectrum scanning was performed between 4000 and 500 cm^−1^. Firstly, 1 mg protein was mixed with 40 mg potassium bromide (KBr) and then ground manually into fine powder. After samples were manually pressed into the disk form, they were placed into the relevant part of the instrument. A spectrum scan was conducted at 1 cm^−1^ resolution at room temperature [30].

#### 2.3.2. Hydrogel Analyses

##### Color

The color measurement was done randomly on the surface of hydrogels by using a chroma meter (Hunter Associates Laboratory, Reston, VA, USA). The values of L*, a*, and b* were used to measure the color. L* = lightness from black to weight 0 to 100, a* = red (+) to green (−) and b* = yellow (+) or blue (−) [31].

##### Water Holding Capacity

WHCs were investigated according to a previous method with some modifications [32]. Briefly, 1 g hydrogel was centrifuged at 3000× *g* for 20 min at 20 °C, and then the centrifuged tube containing the sample was inverted at 45° angles for 30 min to drain the water, and the final mass of the gels was weighed. WHC was noted as a percentage value by dividing the weight of hydrogel after centrifuging by the first weight of hydrogel.

##### Swelling Ratio

The swelling ratios of hydrogels were carried out by cutting hydrogel into a cubic shape with 8 mm diameter and 10 mm length and weight. Cubic hydrogels were kept in a water bath for 30 min at 50 °C. At the end of the period, samples were pulled out of the water bath and drained with filter paper. The swelling ratio was measured according to the following equation [33].

Swelling ratio (%) = W_2_ − W_1_/W_2_ × 100

where W_2_ is the last weight of a hydrogel, and W_1_ is the first weight of a cubic hydrogel sample.

##### Protein Leachability

Briefly, 2 g hydrogel was mixed with 8 mL 0.05 M sodium phosphate buffer (prepared with the mixture of NaH_2_PO_4_ and Na_2_HPO_4_ in the solution) at pH 7.0. After the mixture process was maintained for 2 h at ambient temperature, the samples were exposed to the centrifugation process at 4000 rpm for 15 min. The soluble protein in the supernatant was determined by the Bradford method [34]. Protein leachability was determined as a percentage of protein leaked from hydrogel in 0.05 M sodium phosphate buffer [35].

##### Scanning Electron Microscopy

Hydrogels were freeze-dried. Next, the morphological structures of samples coated with a gold-palladium layer were investigated using scanning electron microscopy (SEM) (ZEISS Sigma 300 Field Emission SEM, Oberkochen, Germany) [36].

##### Textural Behavior

Texture analysis of hydrogels was conducted by using a TA-XT plus texture analyzer (Stable Micro Systems Ltd., Godalming, Surrey, UK) with a cylinder container (35 mm diameter and 30 mm high). The measurements were performed at 1 mm/s pre-test speed, 2 mm/s compression speed, 7 mm compression distance, and 5 g trigger force. All textural parameters, including hardness, gumminess, chewiness, resilience, and springiness, were determined for each hydrogel system [37].

##### Rheological Behavior

The dynamic rheology analysis was conducted by utilizing a rheometer device (Haake MARS II, Thermo Fisher Scientific, Karlsruhe, Germany) with a parallel plate geometry (0.5 mm gap and 20 mm diameter) at 1.00 Hz constant frequency. The oscillation test was carried out to assess storage modulus (G′) and loss modulus (G″). The measurements were performed between 20 and 60 °C [38].

##### Release Behavior

The ability of avocado seed protein-based hydrogels as a bioactive compound delivery system was evaluated for ascorbic acid. Release behavior was carried out according to a previous study [39] with a few modifications. For this purpose, 2 mL of 1.5 M ascorbic acid was injected into hydrogel systems. These systems were transferred into solutions having different natures, namely various pHs (2, 6, and 10), various NaCl concentrations (0.5, 1, and 1.5 M), and various temperatures (25, 50, and 75 °C). Next, the release solution (2 mL) was pipetted every 60 min for 540 min (2 mL of the buffer was added to the release solution after each pipetting). The absorbance of ascorbic acid in the release solution was recorded at 518 nm by utilizing a spectrometer (Model UV-1700, Shimadzu Co., Kyoto, Japan) [40].

#### 2.3.3. Statistical Analysis

All experiments and analyses were carried out with at least three replications. SPSS version 26.0 was used to analyze the data by a one-way analysis of variance (ANOVA). The significant differences were evaluated by Duncan’s test (*p* < 0.05). OriginPro, version 2018 (OriginLab Corp., Northampton, MA, USA), was used for drawing all the graphs.

## 3. Result and Discussion

### 3.1. Physiochemical Properties of Avocado Seed Protein

The approximate composition, including protein, ash, lipid, moisture content, and water activity of ASP, is presented in Table 1. The protein content was slightly different from each other. According to the results, FD has the highest protein content compared with the other counterparts, with values of 67.37, 65.93, 64.81, 62.37, and 62.06% for FD, SD, AD, OD, and VD, respectively. As for moisture content, SD possesses a minimum value (4.22%) compared with the other drying methods. Regarding ash and lipid content, OD and VD have lower values than those of SD, FD, and AD. These differences could be due to drying techniques with different temperatures and durations of process. Moreover, generally, all samples possessed a desirable water activity value in the range of 0.27% to 0.38%.

### 3.2. FT-IR Spectrum for Avocado Seed Protein

**Figure 1:**
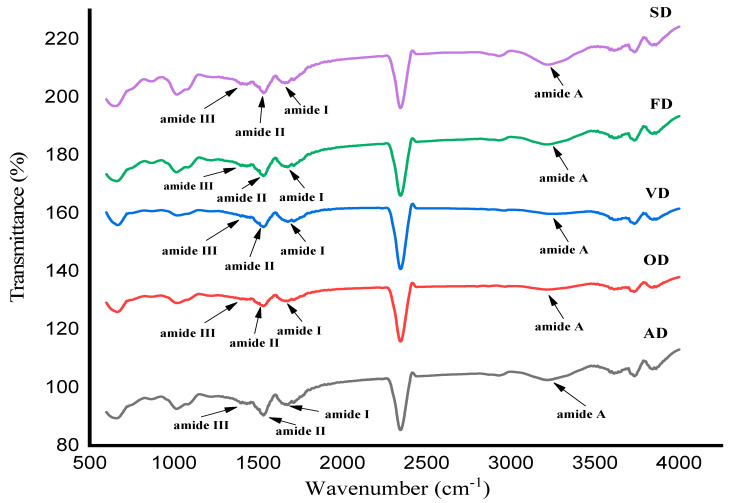
FT-IR spectra of avocado seed proteins prepared by five different drying methods, namely AD, ambient-dried, OD, oven-dried, VD, vacuum-dried, FD, freeze-dried, and SD, spray-dried.

FT-IR spectrum is the most popular technique for determining the secondary structure of proteins, which is responsive to changes in the structure of the peptide chain. The FT-IR spectra of samples at wavelengths between 4000 and 500 cm^−1^ are given in Figure 1. The bands in amide A, amide B, amide I, amide II, and amide III regions to detect the fingerprint of proteins were investigated in this section. The amide A is associated with the N-H stretching vibrations, and the amide B band is related to the stretching vibrations of C-H and NH_2_ stretching vibrations, and both are observed within the spectral range of 3100–3600 cm^−1^ and 2850–2980 cm^−1^, respectively. α-helix, β-sheet, and random coil configurations regarding amide I appear at the wavelengths of 1668–1660 cm^−1^, 1658–1648 cm^−1^, 1648–1640 cm^−1^, and 1625–1610 cm^−1^, respectively. Additionally, the distinctive absorption peaks of proteins, which correspond to amide regions, fell within the range of 1700–1100 cm^−1^ [26]. Amide A is observed in the wavenumber of 3221 cm^−1^ for all samples. The FT-IR spectra of AD, OD, VD, FD, and SD are displayed in the wavenumbers of 1656 cm^−1^ and 1527 cm^−1^, and these wavenumbers are related to amide I (1700–1600 cm^−1^) and amide II (1550–1500 cm^−1^), respectively. The peaks around 1393 cm^−1^ and 1046 cm^−1^ appear in all drying methods, which are related to the amide III and C-H stretching. According to the previous study, certain factors, including heating treatment, long drying time, and organic solvents, display a side effect on the secondary structure of proteins [41]. Proteins were denatured by the constant high temperature utilized in the oven dryer methods. Meanwhile, the freeze-dryer method could cause protein unfolding and aggregation and lessen the number of secondary structures in proteins [42]. Overall, the FT-IR spectra results show that all drying techniques could be utilized for drying proteins, as the amide groups are clearly displayed with slight differences.

### 3.3. Fabrication of Hydrogels Containing the Mixture of Avocado Seed Protein and Locust Bean Gum

This part of the study focused on the usage of ASP in hydrogel systems. Hydrogels, namely AD-ASP + LB, OD-ASP + LB, VD-ASP + LB, FD-ASP + LB, and SD-ASP + LB, were prepared by using mixtures of ASP and LB. These samples were exposed to different analyses, including functional properties, morphological structure, color, textural property, rheological behavior, and release behavior.

#### 3.3.1. Color

One of the most important properties of food products is appearance and color because consumers initially evaluate food products according to these parameters [43]. The values regarding the color of ASP-based hydrogels are illustrated in Table 2. Remarkable differences are detected in the L* results of hydrogel systems with the values of 29.09, 26.62, 28.12, 26.77, and 31.78% for AD-ASP + LB, OD-ASP + LB, VD-ASP + LB, FD-ASP + LB, and SD-ASP + LB, respectively. Similarly, the parameters a* and b* for all hydrogels are not similar to each other (*p* ˂ 0.05), and the highest value for a* and b* is detected in SD-ASP + LB (a*: 3.81 and b: 2.98%). This means that the yellowness value of SD-ASP + LB is superior to the other hydrogels. The possible explanation for this phenomenon is the Maillard reaction between protein and polysaccharides during the synthesis of hydrogels under elevated temperatures.

#### 3.3.2. Water Holding Capacity

WHC is the most important functional property in three-dimensional networks because it is associated with the capacity of hydrogel to hold water [29]. Three-dimensional networks have the ability to absorb additional water [40]. The WHC of ASP-based-hydrogel systems is presented in Table 2. The impact of various drying techniques on this particular functional property is considerable (*p* < 0.05). AD-ASP + LB demonstrated minimum WHC with a value of 64.8%. SD-ASP + LB revealed a comparatively higher ability to retain water with a value of 93.79% when compared with other hydrogels, namely FD-ASP + LB (86.83%), VD-ASP + LB (76.17%), and OD-ASP + LB (72.29%). The findings are consistent with the SEM pictures and mechanical properties exhibited by hydrogels. The gel networks were successfully synthesized using a series of different drying methods, and hydrogel having superior properties was ASP + LB, followed by FD-ASP + LB, VD-ASP + LB, OD-ASP + LB, and AD-ASP + LB when the morphological structures were examined. The results of this study show that ASP dried by SD and FD showed greater tolerance to centrifugal force [41]. Increasing WHC in hydrogel could be related to the increasing number of intermolecular bonds in hydrogel [42]. Moreover, according to the previous study, there is a positive relationship between WHC and hardness [43]. As discussed later, the best and worst hardness were SD-ASP + LB and AD-ASP + LB, respectively. In addition, WHC could be used as an indicator of stability and strength in hydrogels by increasing the value of WHC, indicating that hydrogels could be more stable and strong [5].

#### 3.3.3. Swelling Properties

The swelling properties of the hydrogel are the most essential for cell adhesion, which has a significant impact on cell ventilation and food delivery to the cells [44]. The swelling ratios of ASP-based hydrogels are given in Table 2. Differences between the swelling ratio of the hydrogel are obvious (*p* < 0.05). The superior swelling ratio is detected in SD-ASP + LB with a value of 33.51%, followed by FD-ASP + LB, VD-ASP + LB, OD-ASP + LB, and AD-ASP + LB with a value of 34.1, 23.05, 18.93, and 14.39%, respectively. However, the lowest swelling ratio is identified in OD-ASP + LB (14.39%) due to the partial dissolution. The process of swelling ratio can be understood as an equilibrium between two opposing forces: the stretching of the hydrogel matrix due to the attraction of the solvent by polar and ionic groups and the retraction force exerted by the cross-linked hydrogel structure [45]. According to the previous study, there are some factors that affect the swelling behavior. For example, involving protein and carbohydrates to prepare hydrogel displayed a better swelling ratio than protein alone [44]. The electrostatic repulsive force among two polymers could be another reason behind the swelling ratio [46]. The last factor could involve many hydrophilic groups in hydrogels [47].

#### 3.3.4. Protein Leachability

High protein leachability values are not desired in hydrogel samples [48]. The protein leachability of hydrogel systems is illustrated in Table 2. In this study, the leachabilities of proteins were determined by measuring the amount of proteins leached out from hydrogels (0.05 mol, pH 7.0) to sodium phosphate buffer. The lowest protein leachability is determined in SD-ASP + LB (7.99%) (*p* ˂ 0.05). The maximum protein loss is detected in AD-ASP + LB (24.03%), VD-ASP + LB (19.10%), OD-ASP + LB (17.69%), and FD-ASP + LB (12.14%). The values of protein leachability obtained in our study were in line with a previous study [28]. Whey protein isolates that are enhanced with *Lycium barbarum* polysaccharides at different concentrations and sour cherry seed proteins containing carbohydrates were employed to prepare gelatinous structures. The results obtained in these previous studies indicated that low protein leachability could be related to the presence of an increased number of covalent bonds, which could bring about a strong binding attribute [32,38].

#### 3.3.5. Appearance and Scanning Electron Microscopy Images

**Figure 2:**
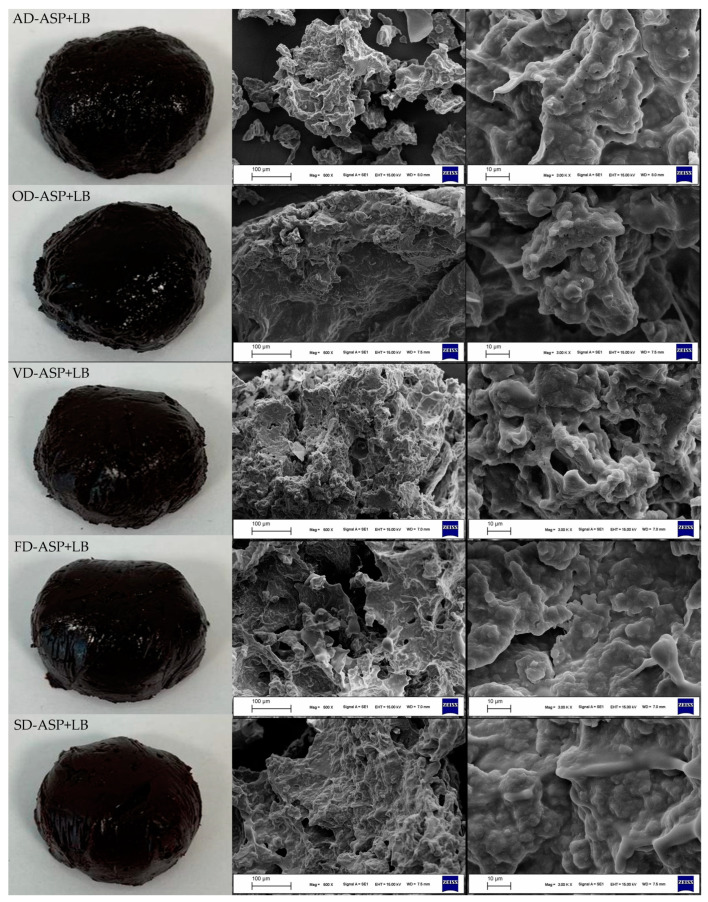
Scanning electronic microscopy of hydrogel systems containing ambient-dried avocado seed protein and locust bean gum (AD-ASP + LB), oven-dried avocado seed protein and locust bean gum (OD-ASP + LB), vacuum-dried avocado seed protein and locust bean gum (VD-ASP + LB), freeze-dried avocado seed protein and locust bean gum (FD-ASP + LB), and spray-dried avocado seed protein and locust bean gum (SD-ASP + LB).

**Figure 3:**
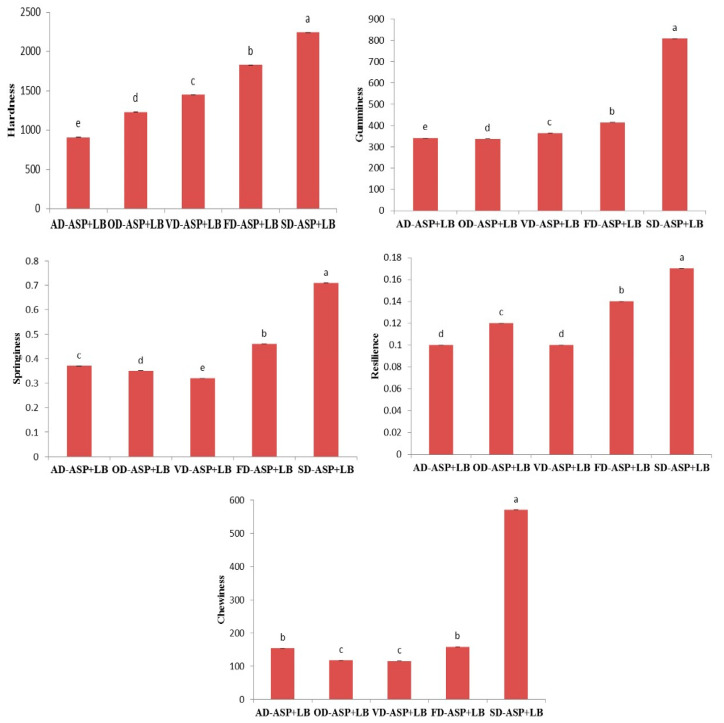
The textural behaviors of hydrogel systems containing ambient-dried avocado seed protein and locust bean gum (AD-ASP + LB), oven-dried avocado seed protein and locust bean gum (OD-ASP + LB), vacuum-dried avocado seed protein and locust bean gum (VD-ASP + LB), freeze-dried avocado seed protein and locust bean gum (FD-ASP + LB), and spray-dried avocado seed protein and locust bean gum (SD-ASP + LB). The results were displayed as mean ± standard deviation and dissimilar letters in the same graph among the samples show significant differences between samples (*p* ˂ 0.05).

**Figure 4:**
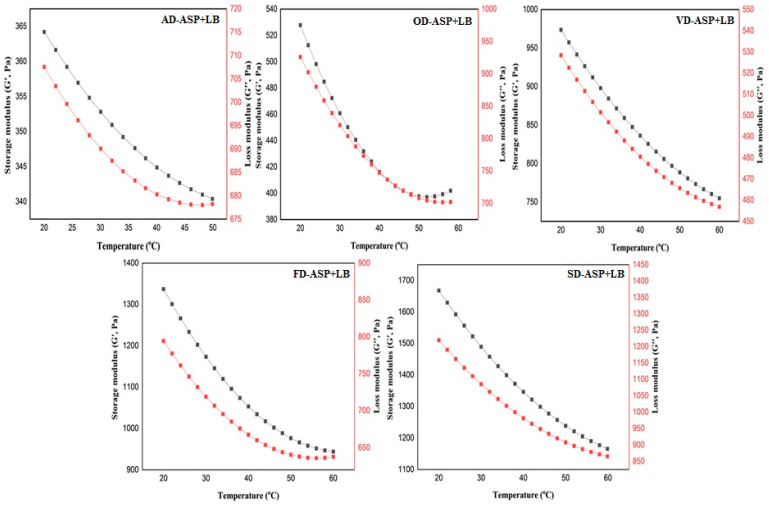
Rheological behaviors of hydrogel models containing ambient-dried avocado seed protein and locust bean gum (AD-ASP + LB), oven-dried avocado seed protein and locust bean gum (OD-ASP + LB), vacuum-dried avocado seed protein and locust bean gum (VD-ASP + LB), freeze-dried avocado seed protein and locust bean gum (FD-ASP + LB), and spray-dried avocado seed protein and locust bean gum (SD-ASP + LB).

The appearance and SEM images of the hydrogel systems are illustrated in Figure 2. Among samples, alterations in terms of visual characteristics were obvious. All hydrogel systems possessed a three-dimensional polymer network. However, the interaction level of polymer chains was not the same when SEM images were examined. In other words, considerable changes were observed in the morphological structures of ASP-based hydrogels, depending on the specific drying procedures. Comparatively, non-homogeneous porous structures in the SEM images of AD-ASP + LB, OD-ASP + LB, and VD-ASP + LB were dominant. In contrast, these structures disappeared in the SD-ASP + LB and FD-ASP + LB. Denser (more compact) was determined in these hydrogel systems. The observed phenomena could be attributed to more hydrogen bonds and higher electrostatic interactions between proteins and carbohydrates in the hydrogel matrix [48]. More hydrogen bonds and higher electrostatic interactions in hydrogel systems lead to the formation of greater rigid structures [37]. In order to obtain a desirable three-dimensional structure, it is essential that the interactions between the polymers forming the system are high [49]. Such systems possess a robust structure and exhibit superior functional properties [38]. These phenomena were supported by the current findings. The values in terms of functional properties and mechanical behavior for SD-ASP + LB and FD-ASP + LB were ahead of those of other counterparts when Table 2 and Figure 3 and Figure 4 were examined in detail.

#### 3.3.6. Textural Behavior

The hardness of samples is measured from the first compression to the maximum force. Resilience is obtained by the ratio of the force upstroke to the down stock from the first compression [32]. The springiness is measured by calculating the distance ratio of the input force into the hydrogel during the second compression from the first compression. Gumminess could be utilized as an indication to display the situation of a semisolid sample and determined by calculation (hardness × cohesiveness) [50]. Chewiness can be used to measure the situation of a solid sample and determine by calculation (hardness × cohesiveness × springiness) [51]. The network structure of protein hydrogel prepared by heating is mostly dependent upon the balance between the force of attractiveness and repulsiveness among the molecular denaturation of proteins during aggregation protein [52]. These parameters are one of the most important properties of samples, including hydrogel, in terms of consumer preference. The findings regarding the textural parameters, namely hardness, gumminess, chewiness, resilience, and springiness of hydrogel systems, are shown in Figure 3. The values regarding these textural parameters of hydrogels prepared with ASP dried by various methods are not similar to each other (*p* < 0.05). There are significant differences between hydrogels in terms of hardness (*p* < 0.05). The maximum hardness value among the hydrogels was determined in SD-ASP + LB, followed by FD-ASP + LB, VD-ASP + LB, OD-ASP + LB, and AD-ASP + LB. Two main reasons can improve the hardness of hydrogel. First of all, the pH value of hydrogel is raised above the isoelectric point. Secondly, hardness could be enhanced by increasing the solid content during the preparation of hydrogel by increasing the concentration of protein or carbohydrates [44]. There are some studies corresponding to these findings, and they are investigating that basic amino acids have an effect on the hydrogel properties and during the electrostatic interactions, basic amino acids strongly bind to the protein-charged residues as a result of changes in the structure and thermal characteristics of proteins [53,54,55,56]. The gumminess, chewiness, resilience, and springiness showed similar patterns with hardness. In addition, SD-ASP + LB and FD-ASP + LB have the maximum value among other hydrogel samples in terms of gumminess, chewiness, resilience, and springiness (*p* < 0.05). All findings regarding appearance, SEM images, functional properties, and rheological behavior of hydrogels are supported by the dataset.

The data are given as mean ± standard deviation, and the different letters in the same graph show the differences between the samples (*p* ˂ 0.05).

#### 3.3.7. Dynamic Rheology Behavior

Storage modulus (G′) and loss modulus (G″) are two parameters responsible for the elasticity and viscous behavior of hydrogel, respectively. The dynamic oscillation test was conducted to measure the impact of cross-linked between different dried proteins and locust beans based on plant protein hydrogel. The impact of temperature (from 20 °C to 60 °C) on rheological behavior (G′ and G″) of hydrogels is shown in Figure 4. The curves related to the G′ and G″ of hydrogel displayed similar patterns, and the values of both moduli were gradually reduced by rising temperature. This indicates that three-dimensional structures of hydrogels were converted from a solid-like gel to a liquid-like solution [38,57]. Figure 4 clearly displays that there are significant differences among the rheological behavior of hydrogels when their G′ and G″ values are examined. The superior G′ value is detected in SD-ASP + LB, followed by FD-ASP + LB, OD-ASP + LB, VD-ASP + LB, and AD-ASP + LB. The result clearly proves that the SD-ASP + LB possesses the stiffest internal structure. More junction zones in this hydrogel system, thanks to more electrostatic complexes and H-bonding, could be given a reason for the robust structure. Furthermore, according to the results, the value of G′ is higher than G″ for all hydrogel samples, which means that hydrogels of three-dimensional structure are forming at an acceptable level [47]. Additionally, the variations in the values G′ and G″ show the formation of hydrogel models with elasticity-dominated rather than viscous systems. Ultimately, the results indicate that, according to the elastic G′ and viscous G″, drying protein by SD and FD are better drying methods for forming hydrogel than other dryers.

### 3.4. Release Behavior

**Figure 5:**
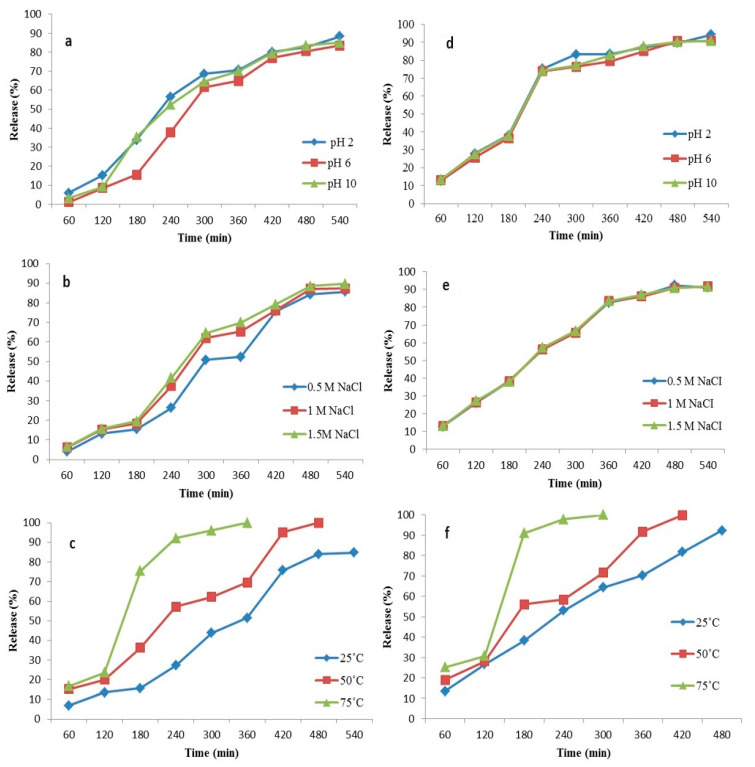
Release behavior of ascorbic acid from hydrogels system prepared by spray-dried avocado seed protein and locust bean gum (**a**–**c**) and spray-dried avocado seed protein alone (**d**–**f**).

Ascorbic acid is known as vitamin C, which is a water-soluble compound. It is an organic compound with a white color and crystalline structure. This structure could be extracted from fresh fruits. Ascorbic acid, a functional compound, has a positive impact on human health, and its main role in the human body is to activate many enzymes in the liver, which are related to the hormones, nervous system, and detoxification by drug compounds. Additionally, this functional compound exhibits antioxidant and anti-inflammatory behavior and possesses a potential for the treatment of many diseases, including cancer, neurodegenerative disorders, and cardiovascular diseases [15]. However, ascorbic acid should be encapsulated into various matrices to improve stability and bioavailability before being utilized in the mentioned systems. One of the most effective approaches to fulfill this criterion is the integration of these entities inside the hydrogel matrix using either physical entrapment or chemical cross-linking methods, which are implemented during the synthesis of three-dimensional polymeric networks [58]. In this study, considering other findings, superior results are obtained in SD-ASP + LB. Therefore, ascorbic acid was injected into this hydrogel model, and the amount of this compound leaked out of the gel to the surrounding solution was monitored for 540 min at ambient temperature. A gel system containing spray-dried protein alone was selected as a control. Figure 5 shows the diffusion rate of bioactive compounds from the interior of the hydrogel matrix to three different surrounding environments, including different pH, molarity of NaCl, and temperature. In terms of pH, the release value of ascorbic acid in SD-ASP + LB is minimum (1.1%) at pH 6 compared with pH 10 (3.13%) and pH 2 (5.96%) during the first 60 min, as illustrated in Figure 5a. Ascorbic acid in both samples sharply leaked out, and the diffusion rate reached ~40% in all pH parameters from 120 min to 240 min. As a result, pH 6.0 shows the minimum release of bioactive compounds in the hydrogel system. Figure 5b displays the amount of the bioactive structure leaked out from the interior of the hydrogel matrix to the surrounding solution (0.5, 1, and 1.5 mol NaCl). The diffusion level of ascorbic acid to all NaCI solutions is the highest between 180 and 300 min. Moreover, the lowest leakage for SD-ASP + LB is detected in 0.5 mol NaCl. As for temperature, the minimum release for both hydrogel systems is determined to be 25 °C. On the other hand, the diffusion rate of ascorbic acid increased due to burst release after 120 min at 50 and 75 °C. The abrupt cumulative transfer of bioactive chemicals out of the hydrogel is caused by a burst release mechanism [59]. This method might lead to a quick depletion of the molecule and decreased efficiency over time, which makes it undesirable in delivery systems. As a result, these systems could be avoided in a variety of applications such as cosmetics, medicines, and food preservation [60]. Among the five different avocado seed proteins, hydrogels constituted with spray-dried protein displayed the best release behavior due to the presence of a positive relation between pores (swelling ratio) and release. The previous study reported that loading more ascorbic acid solution into the sample with a minimum swelling ratio could disintegrate the hydrogel matrix, and this could be the reason for the physical interruption of the three-dimensional network [57].

## 4. Conclusions

Several studies have been carried out to enhance the diverse quality attributes of plant-based proteins. These studies particularly focus on their physicochemical and functional properties. Similarly to these approaches, the current study investigated the applicability of drying methods for increasing the physicochemical and functional characteristics of a novel fruit protein (ASP) produced from fruit processing by-products. Drying techniques had a significant effect on the physicochemical and chemical structure of ASP. The results showed that SD and FD were better techniques compared with the other dried methods, especially in the protection of secondary structures. This means that SD and/or FD, compared to their counterparts, were ahead in the production of proteins with intended functional features for industrial processes. The desirable impact of the mentioned techniques, especially SD, was obvious in the characteristic properties (appearance, microstructure, functionality, texture property, and rheology behavior) of hydrogel systems. In other words, the superior findings in terms of functional properties, namely WHC, swelling ratio, and protein leachability, were obtained in SD-ASP + LB and FD-ASP + LB compared with the other hydrogels. Similarly, textural behavior results showed that robust three-dimensional structure was detected in SD-ASP + LB and FD-ASP + LB, followed by VD-ASP + LB, OD-ASP + LB, and AD-ASP + LB. These findings, supported by the rheological behavior of hydrogels and similar hierarchical order, were valid for G′, indicating that gel systems with the strongest network structure were SD-ASP + LB and FD-ASP + LB. Moreover, the transformation from a solid-like gel to a liquid-like solution with an increase in temperature appeared in all hydrogels. A realistic strategy for enhancing the ability of ASP-based hydrogel systems as the bioactive compound delivery was to incorporate LB into them. In future studies, various drugs will be loaded into SD-ASP + LB, and their effectiveness will be investigated in in vitro and/or in vivo conditions.

## Figures and Tables

**Table 1:** Chemical composition of the avocado seed protein prepared by five different drying methods.

Parameters	AD	OD	VD	FD	SD
Protein content	64.81 ± 0.17 ^c^	62.37 ± 0.26 ^d^	62.06 ± 0.53 ^d^	67.37 ± 0.61 ^a^	65.93 ± 0.17 ^b^
Moisture content	5.35 ± 0.07 ^a^	5.35 ± 0.08 ^a^	4.88 ± 0.04 ^b^	4.69 ± 0.08 ^c^	4.22 ± 0.04 ^d^
Lipid content	2.4 ± 0.14 ^ab^	2.25 ± 0.07 ^b^	2.15 ± 0.07 ^b^	2.6 ± 0.14 ^a^	2.7 ± 0.15 ^a^
Ash content	8.7 ± 0.14 ^a^	8.7 ± 0.32 ^a^	8.55 ± 0.35 ^a^	8.8 ± 0.21 ^a^	8.8 ± 0.28 ^a^
Water activity	0.38 ± 0.00 ^a^	0.27 ± 0.01 ^e^	0.35 ± 0.00 ^b^	0.34 ± 0.00 ^c^	0.32 ± 0.00 ^d^

Mean ± standard division (*n* = 3). Different letters (^a–e^) are significantly different in the same row (*p* ˂ 0.05). ASP: avocado seed protein. AD: ambient-dried. OD: oven-dried. VD: vacuum-dried. FD: freeze-dried. SD: spray-dried.

**Table 2:** Color and functional properties of hydrogels.

	Color					
	L*	a*	b*	Water Holding Capacity (%)	Swelling Ratio (%)	Protein Leachability (%)
AD-ASP + LB	29.09 ± 0.05 ^b^	2.17 ± 0.22 ^b^	1.34 ± 0.13 ^b^	64.8 ± 0.72 ^e^	14.39 ± 0.15 ^d^	24.03 ± 0.55 ^e^
OD-ASP + LB	26.62 ± 0.23 ^c^	2.63 ± 0.31 ^b^	1.88 ± 0.02 ^b^	72.29 ± 0.48 ^d^	18.93 ± 0.21 ^c^	17.69 ± 0.13 ^c^
VD-ASP + LB	28.12 ± 0.33 ^b^	2.61 ± 0.1 ^b^	1.57 ± 0.02 ^b^	76.17 ± 2.33 ^c^	23.05 ± 0.11 ^b^	19.1 ± 0.25 ^d^
FD-ASP + LB	26.77 ± 0.99 ^c^	2.38 ± 0.23 ^b^	1.78 ± 0.44 ^b^	86.83 ± 0.18 ^b^	34.1 ± 1.86 ^a^	12.14 ± 0.13 ^b^
SD-ASP + LB	31.78 ± 0.04 ^a^	3.81 ± 0.15 ^a^	2.98 ± 0.05 ^a^	93.79 ± 0.96 ^a^	33.51 ± 0.42 ^a^	7.99 ± 0.67 ^a^

Each datum represents the mean of three replications ± standard deviation. The small letters in the same column (^a–e^) show significant differences between samples (*p* ˂ 0.05). Hydrogel systems containing ambient-dried avocado seed protein and locust bean gum (AD-ASP + LB), oven-dried avocado seed protein and locust bean gum (OD-ASP + LB), vacuum-dried avocado seed protein and locust bean gum (VD-ASP + LB), freeze-dried avocado seed protein and locust bean gum (FD-ASP + LB) and spray dried avocado seed protein and locust bean gum (SD-ASP + LB).

## Data Availability

The datasets from the current study are available from the corresponding author upon reasonable request.
